# Agreement of left ventricular mass in steady state free precession and delayed enhancement MR images: implications for quantification of fibrosis in congenital and ischemic heart disease

**DOI:** 10.1186/1471-2342-10-4

**Published:** 2010-01-24

**Authors:** Sigurdur S Stephensen, Marcus Carlsson, Martin Ugander, Henrik Engblom, Goran Olivecrona, David Erlinge, Hakan Arheden

**Affiliations:** 1 Department of Radiology and Physiology, Lund University and Lund University Hospital, Lund, Sweden; 2 Department of Pediatric Cardiology, Lund University and Lund University Hospital, Lund, Sweden; 3 Department of Cardiology, Lund University and Lund University Hospital, Lund, Sweden

## Abstract

**Background:**

Left ventricular mass (LVM) is used when expressing infarct or fibrosis as a percentage of the left ventricle (LV). Quantification of LVM is interchangeably carried out in cine steady state free precession (SSFP) and delayed enhancement (DE) magnetic resonance imaging (MRI). However, these techniques may yield different LVM. Therefore, the aim of the study was to compare LVM determined by SSFP and DE MRI in patients and determine the agreement with these sequences with ex vivo data in an experimental animal model.

**Methods:**

Ethics committees approved human and animal studies. Informed written consent was obtained from all patients. SSFP and DE images were acquired in 60 patients (20 with infarction, 20 without infarction and 20 pediatric patients). Ex vivo MRI was used as reference method for LVM in 19 pigs and compared to in vivo SSFP and DE.

**Results:**

LVM was greater in SSFP than in DE (p < 0.001) with a bias of 5.0 ± 6.7% in humans (r^2 ^= 0.98), and a bias of 7.3 ± 6.7% (p < 0.001) in pigs (r^2 ^= 0.83). Bias for SSFP and DE images compared to ex vivo LVM was -0.2 ± 9.0% and -7.7 ± 8.5% respectively.

**Conclusions:**

LVM was higher when measured with SSFP compared to DE. Thus, the percentage infarction of the LV will differ if SSFP or DE is used to determine LVM. There was no significant difference between SSFP and ex vivo LVM suggesting that SSFP is more accurate for LVM quantification. To avoid intrinsic error due to the differences between the sequences, we suggest using DE when expressing infarct as a percentage of LVM.

## Background

Myocardial infarct size influences patient prognosis [1] and left ventricular (LV) remodeling [2]. Therefore, quantification of infarct size as a percentage of the LV myocardium is of great interest in preclinical and clinical trials as well as in the clinical setting [3,4]. Magnetic resonance imaging (MRI) has been established as the in vivo reference method for quantification of left ventricular mass (LVM) and myocardial infarction [5-10]. Myocardial infarction is quantified in delayed enhancement (DE) MRI where infarcted myocardium is hyperenhanced [11]. Furthermore, DE-MRI has recently been shown to have the capability to detect fibrosis of the right ventricle in patients with surgically corrected congenital heart disease [12-14]. The percentage infarcted or fibrotic myocardium of the LV is most often calculated as the hyperenhanced myocardium in the DE images divided by LVM in the steady state free precession (SSFP) cine images [15-17]. However, investigators have also calculated the percentage of hyperenhanced myocardium with LVM obtained from DE images [18,19]. Since the assessment of LV dimensions and LVM differ between SSFP and gradient-echo (GRE) images [20] the LVM quantification may also differ between DE and SSFP and this will impact the amount of infarction as a percentage of LVM. Recently, a study showed good agreement between DE and SSFP in determining LVM [21] but is not clear if these results are reproducible using a MR scanner from a different vendor and another patient population. Furthermore, in a clinical study there is no reference method to determine which sequence gives the correct LVM. High resolution ex vivo MRI in animal models however, gives the ability to measure LVM with high resolution and accuracy [6,22,23] and can be used as a reference method for LVM. Therefore, the aim of the present study was 1) to investigate if LVM determined by SSFP images or DE images differ in human subjects and 2) to use ex vivo cardiac MR images in a porcine infarct model as a reference method to determine which in vivo sequence gives the most accurate LVM.

## Methods

### Study population

The local ethics committee approved the study and written informed consent was obtained from all patients prior to examination. The animal studies were approved by the local animal ethics committee and the study conformed to the Guide for the Care and Use of Laboratory Animals, US National Institute of Health (NIH Publication No 85-23, revised 1996). Sixty patients were included in the study; twenty adults with no signs of myocardial infarct on DE-MRI, 20 adults with myocardial infarct on DE-MRI and 20 children; five of whom underwent cardiac MRI because of suspected myocarditis but had normal findings, and 15 children who had undergone heart surgery for tetralogy of Fallot, but had no signs of fibrosis of the LV. Patient details are presented in Table 1. All subjects underwent cardiac MRI in the supine position and images were acquired during end-expiratory breath hold.

**Table 1:** Patient characteristics

	Children	Patients without MI	Patients with MI
*N*	20	20	20
Age in years (range)	12 ± 3 (9-17)	59 ± 8 (42-76)	70 ± 11 (42-82)
Females/Males	11/9	9/11	6/14

MI, myocardial infarction

For comparison of in vivo and ex vivo LVM, nineteen domestic male and female pigs, weighing 40-50 kg, were imaged. After overnight fasting with free access to water the animals were premedicated with ketamine 15 mg/kg (Ketaminol, Intervet, Danderyd, Sweden) and xylazin 2 mg/kg intramuscularly (Rompun, Bayer AG, Leverkusen, Germany). Anesthesia was induced with thiopental 12.5 mg/kg (Pentothal, Abbot, Stockholm, Sweden) and infusion of fentanyl (Fentanyl, Pharmalink AB, Stockholm, Sweden) was started. Cardiac MRI was performed in vivo after experimentally induced myocardial infarction by inflation of angioplasty balloon in the left anterior descending coronary artery distal to the first diagonal branch. After the animals were sacrificed, their hearts were explanted and imaged ex vivo according to a previously described protocol [24,25]. See below for details.

### MR imaging

A 1.5 Tesla MRI scanner with a 5-element cardiac synergy coil was used for all in vivo and ex vivo studies (Philips Intera CV, Philips, Best, the Netherlands). SSFP and DE images were acquired covering the left ventricle from the base to the apex in the short-axis plane. Inversion time was set to null viable myocardium. Typical MRI sequence parameters for SSFP were: echo time 1.5 ms, repetition time 3 ms, flip angle 60°, slice gap 0 mm, slice thickness 8 mm (6 mm in children), inplane resolution 1.1 × 1.1 to 1.6 × 1.6 mm and SENSE factor 2.

An inversion recovery GRE-sequence was used to obtain DE images in the short axis plane 10-20 minutes after intravenous administration of 0.2 mmol/kg gadolinium based MR contrast media (Magnevist, Bayer Pharma, Berlin, Germany). Acquisition time for DE was mid diastole. For DE the sequence parameters were: echo time 1.3 ms, repetition time 4 ms, flip angle 15°, slice gap 0 mm and slice thickness 8 mm, inplane resolution 0.9 × 0.9 to 1.6 × 1.6 mm.

In vivo imaging of the animals was performed using the same SSFP and DE imaging parameters as in patients. Following euthanasia the animal hearts were removed, the atria excised and the ventricles filled with deuterated water. Ex vivo T1-weighted (T1w) MR imaging was performed with a quadrature head coil covering the left ventricle from the base to the apex, typically resulting in 150 image slices per heart. The sequence parameters were: echo time 3.2 ms, repetition time 20 ms, flip angle 70°, slice gap 0 mm and slice thickness 0.5 mm, inplane resolution 0.5 × 0.5.

All MR images were evaluated by two blinded observers using freely available software (Segment 1.688, http://segment.heiberg.se). The LVM was quantified by outlining the endo- and epicardium of the left ventricle in the SSFP, DE and ex vivo images and multiplying the resulting volume by 1.05. Outlining of in vivo images was carried out in end diastole and end systole and the mean value calculated. The papillary muscles were included in the LVM as previously suggested and used in reference values for LVM [26].

### Statistical analysis

Continuous variables were presented as mean ± SD and range. Pearson's correlation was used to determine the relationship between different techniques in determining LVM. Two-tailed paired t-test was used to detect differences in LVM between the two techniques. A p-value below 0.05 was considered significant. Bias between LVM using SSFP and DE was calculated according to Bland-Altman and presented as the mean difference ± SD. Bias between ex vivo LVM and SSFP or DE was calculated using ex vivo as reference LVM. Interobserver variability was calculated for all subjects and presented as mean ± SD.

## Results

**Figure 1:**
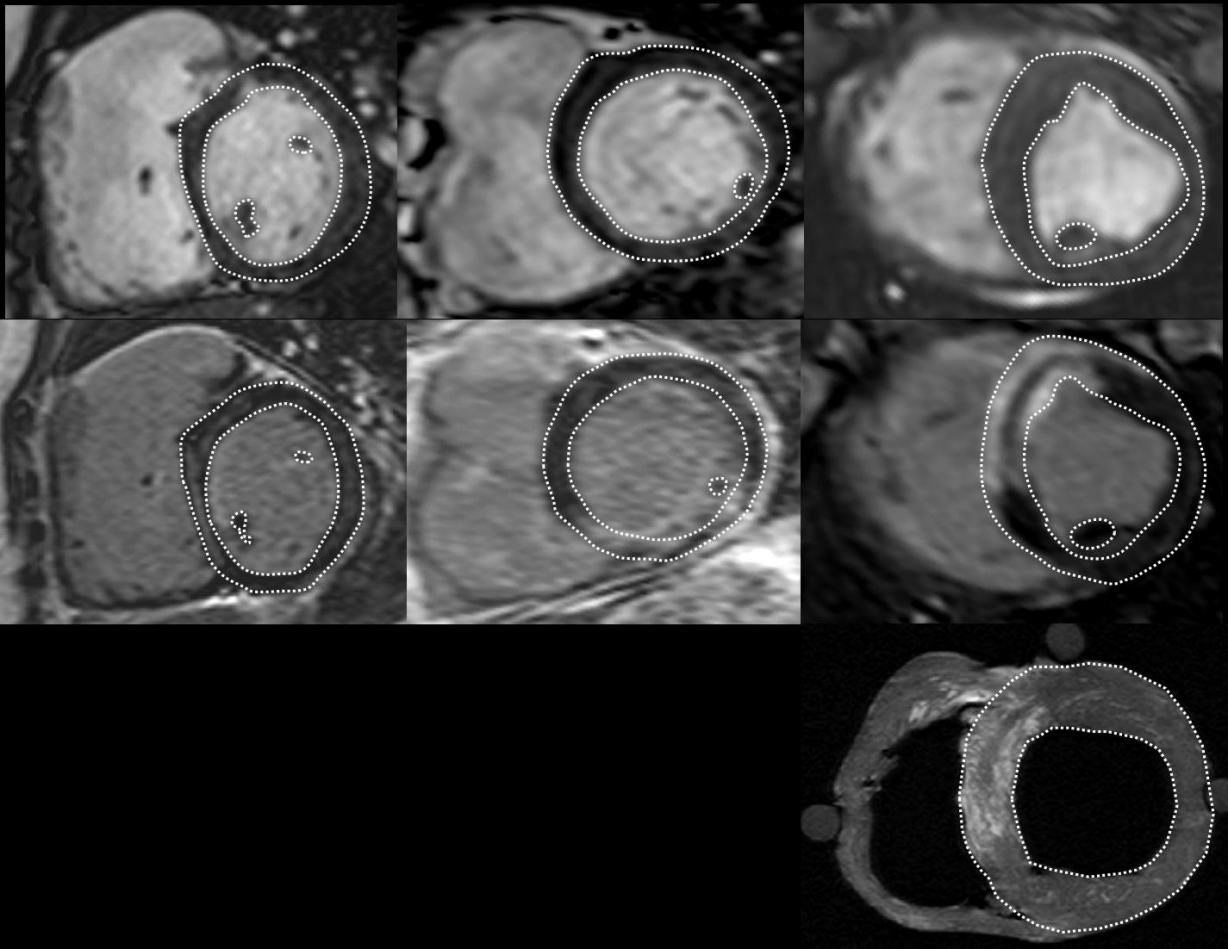
**Short axis images of the heart on SSFP (top row) and DE (middle row) sequences in a child (left column), an adult (middle column) and a pig (right column)**. Bottom row shows the ex vivo T1w image of the pig heart.

Representative short axis MR images of the LV in patients and pigs are illustrated in Figure 1. MR images with corresponding delineations of the LVM from the three sequences used in the study, namely SSFP, DE and ex vivo T1w are shown. LVM assessment on DE and SSFP in the three patient groups as well as the in-vivo animals is presented in table 2.

**Table 2:** LVM measurements on DE and SSFP in the three study groups and the pigs

	Children	Patients without MI	Patients with MI	Total	Pigs
LVM on DE in grams (range)	73.7 ± 31.8 (37.4-151.6)	132.4 ± 32.8 (93.5-220.2)	155.4 ± 44.0 (74.6-275.1)	120.5 ± 50.6 (37.4-275.1)	93.6 ± 14.5 (71.4-127.2)
LVM on SSFP in grams (range)	79.4 ± 34.0 (37.3-162.7)	139.7 ± 35.7 (97.6-242.8)	159.5 ± 45.8 (77.1-285.4)	126.2 ± 52.1 (37.3-285.4)	100.9 ± 15.9 (80.9-133.6)
Bias	7.2 ± 8.4%	5.2 ± 5.0%	2.5 ± 5.5%	5.0 ± 6.7%	7.5 ± 6.9%

LVM, left ventricular mass; DE, delayed enhancement; SSFP, steady state free precession; MI, myocardial infarct

### Patients

**Figure 2:**
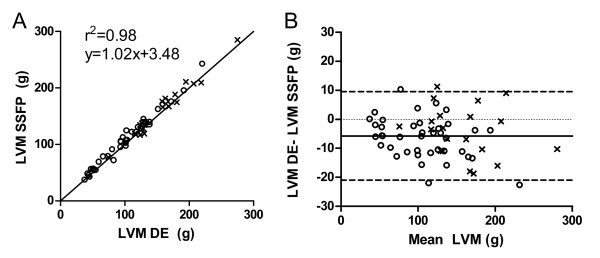
**Pearson's correlation and Bland-Altman analysis of LVM measured on SSFP and DE in all 60 patients**. Open circles signify the 40 patients without infarcts and crosses signify the 20 patients with infarcts.

Myocardial mass measured with SSFP images (126.2 ± 52.1 g, range 37.3-285.4 g) was higher compared to DE images (120.5 ± 50.6 g, range 37.4-275.1 g, p < 0.001). The bias for LVM measurement between SSFP and DE images was 5.0 ± 6.7% (figure 2). However, the correlation between LVM determined on SSFP and DE images was high (r^2 ^= 0.98, p < 0.001, LVMSSFP = 1.0(LVMDE)+3.5). The difference in LVM between SSFP and DE remained if the 20 patients with myocardial infarct (MI) were excluded (109.6 ± 46.7 g vs. 103.1 ± 44.2 g respectively, p < 0.001, r^2 ^= 0.98, p < 0.001, LVMSSFP = 1.0(LVMDE)+1.7). LVM in the 40 subjects without MI was higher by 6.2 ± 6.9% (p < 0.001) when measured with SSFP compared to DE. In the 20 patients with MI the bias was 2.5 ± 5.5% (p < 0.001).

### Animals

**Figure 3:**
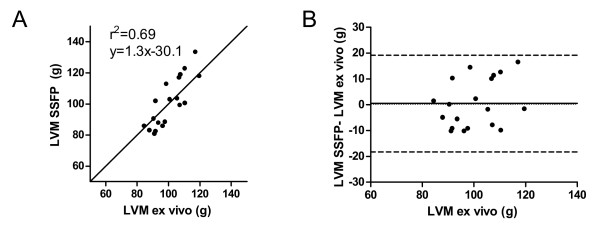
**Pearson's correlation and Bland Altman analysis of LVM in pigs measured on SSFP and ex vivo**.

**Figure 4:**
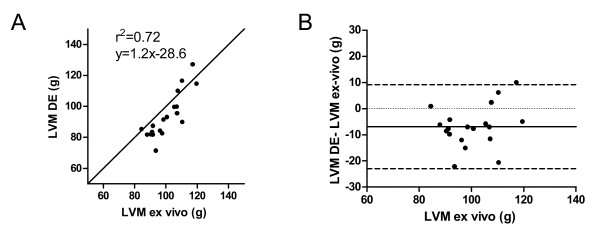
**Pearson's correlation and Bland Altman analysis of LVM in pigs measured on DE and ex vivo**.

LVM in pigs was also higher when measured with SSFP (100.9 ± 15.9 g, range 80.9-133.6 g) compared to DE (93.6 ± 14.5 g, range 71.4-127.2 g, p < 0.001). The bias for LVM measurement between SSFP and DE images was 7.5 ± 6.9%. There was a moderate correlation (r^2 ^= 0.69, p < 0.001, LVMSSFP = 1.3(LVMex-vivo)+30.1) between LVM measured on SSFP and ex vivo T1w MRI (100.4 ± 10.1 g, range 84.4-119.5 g) and the difference between the methods was not significant (0.2 ± 9.0%, p = 0.82), (figure 3). In contrast, LVM was higher on ex vivo MRI compared to DE (7.7 ± 8.5%, p < 0.01) although the correlation was similar (r^2 ^= 0.72, p < 0.01, LVMDE = 1.2(LVMex-vivo)-28.6), (figure 4). Interobserver variability for the measurements of LVM in humans and pigs was 2.9 ± 6.6% for SSFP and 2.7 ± 6.7% for DE.

## Discussion

This study has shown that SSFP MRI yields significantly higher LVM compared to DE MRI in patients. This difference was also confirmed in animals where LVM determined in vivo on SSFP most closely resembled LVM measured on high resolution ex vivo MRI. Quantification of infarct size as percentage of LVM on MRI will be influenced by this difference in LVM between the SSFP and DE sequences.

Quantification of infarct size is an important prognostic factor in patients with ischemic heart disease [1,2]. The extent of fibrotic myocardium is also of value in patients who have had surgery for congenital heart disease, such as tetralogy of Fallot [14] as it affects right ventricular function in adulthood and is related to restrictive physiology, exercise intolerance and arrhythmias [27]. In this study we have evaluated two methods used to quantify LVM when calculating infarct as percentage of LVM and showed that they are not interchangeable. The LVM was higher when measured with SSFP cine sequences compared to DE sequences. Therefore the proportion of infarct of the LVM will differ depending if SSFP images or DE images are used to determine LVM. The findings of the current study are in contrast with previous findings by Grothues et al who found good correlation between the two techniques but, opposite to our results, generally lower values of myocardial mass when measured with SSFP [21]. Possible reasons for this discrepancy may be due to differences in 1) MRI scanners, 2) patient populations and 3) delineation technique. Grothues et al studied patients with a first time documented myocardial infarct but we also included patients with no infarction and children. In the present study the bias between SSFP and DE was lower in patients with MI compared to those without MI, suggesting that parts of the discrepancy between the two studies can be explained by the different patient populations. Furthermore, our results were confirmed in an experimental setting with high resolution ex vivo imaging.

In the clinical setting the difference of 5.0% ± 6.7% may be considered negligible and this is supported by the high correlation between the methods. However, it is important to be aware of this discordance and use the same acquisition method in the follow-up of patients, for example when following infarct shrinkage [28-30]. Due to the differences between sequences there is a risk of introducing intrinsic error, when calculating infarct size using variables from two different sequences. Indeed, in the study of Grotheus et al, LVM measured on a GE scanner revealed higher LVM on DE than SSFP by 2.4 ± 3.5% [21]. The difference in LVM may thus become even greater when comparing results from different types of MRI scanners and using different sequences. Therefore, we suggest using DE for the quantification of LVM and the infarcted area when presenting percentage infarct quantification. When using automated quantification of infarct size, which is recommended for scientific purposes [10], delineation of the myocardium in DE images is necessary. Therefore, this does not add extra delineations of the myocardium. However, the high accuracy and precision of the SSFP sequence, compared to ex vivo imaging, supports the use of SSFP sequences when determining LVM in the clinical setting. In any case, the methodology for LVM measurements must be determined and should be stated when performing clinical or preclinical studies. The presence of infarction may influence the quantification of LVM on DE, since the delineation of the endocardial border can be difficult. However this did not seem to affect the results of the present study, as the LVM was smaller in DE compared to SSFP both in hearts with and without MI. In pigs we considered the ex vivo images as the reference standard that best estimates the real LVM. There was no significant difference between the SSFP images and the ex vivo images and therefore one can assume that the SSFP images give better assessment of LVM, while it is slightly underestimated in the DE images. A possible explanation for this discrepancy is that SSFP sequences give better contrast between papillary muscles and blood. In the DE sequences the papillary muscles and trabeculations are not as clear, running the risk that they are not included in the delineation and measurement of myocardial mass. This can also be influenced by the time of imaging after contrast administration which can affect the myocardial to blood contrast.

### Limitations

We included a diverse group of subjects with a wide range of age and myocardial mass. However, all patients were examined with the same type of scanner (1.5 T Philips Intera CV) and DE sequence and it is possible that other vendors and sequences may yield different results. We did not measure the weight of the LVM in the animals but rather used ex vivo high resolution MRI to obtain the LVM. This could have provided an independent variable for further valuation of the results. The myocardial-to-blood contrast-to-noise ratio was not measured for each subject. However, when acquiring the DE imaging great care was taken to choose the right inversion time to null all viable myocardium. These factors may be considered when performing further studies on this subject.

## Conclusions

The present study has shown a higher LVM on SSFP compared to DE MRI in patients and animals and that SSFP agrees closely with ex vivo high resolution MRI. We suggest using SSFP when quantifying LVM only, but using mass from DE when presenting infarction as percentage of LVM. The difference in LVM on different MRI sequences found in the present study needs to be considered in future guidelines for quantification of infarct size.

## Competing interests

The authors declare that they have no competing interests.

## Authors' contributions

SSS analyzed all data, and drafted the manuscript. MC analyzed all data and participated in the study design. MU, HE, DE and GO designed and performed the experimental studies. HA conceived the study and participated in its design. All authors read and approved the final manuscript.

## Pre-publication history

The pre-publication history for this paper can be accessed here:

http://www.biomedcentral.com/1471-2342/10/4/prepub
